# Both Group 4 Capsule and Lipopolysaccharide O-Antigen Contribute to Enteropathogenic *Escherichia coli* Resistance to Human α-Defensin 5

**DOI:** 10.1371/journal.pone.0082475

**Published:** 2013-12-04

**Authors:** Jenny-Lee Thomassin, Mark J. Lee, John R. Brannon, Donald C. Sheppard, Samantha Gruenheid, Hervé Le Moual

**Affiliations:** 1 Department of Microbiology and Immunology, McGill University, Montreal, Quebec, Canada; 2 Microbiome and Disease Tolerance Centre, McGill University, Montreal, Quebec, Canada; 3 Faculty of Dentistry, McGill University, Montreal, Quebec, Canada; Universidad Nacional de La Plata., Argentina

## Abstract

Enteropathogenic and enterohemorrhagic *Escherichia coli* (EPEC and EHEC) are food-borne pathogens that colonize the small intestine and colon, respectively. To cause disease, these pathogens must overcome the action of different host antimicrobial peptides (AMPs) secreted into these distinct niches. We have shown previously that EHEC expresses high levels of the OmpT protease to inactivate the human cathelicidin LL-37, an AMP present in the colon. In this study, we investigate the mechanisms used by EPEC to resist human α-defensin 5 (HD-5), the most abundant AMP in the small intestine. Quantitative PCR was used to measure transcript levels of various EPEC surface structures. High transcript levels of *gfcA*, a gene required for group 4 capsule (G4C) production, were observed in EPEC, but not in EHEC. The unencapsulated EPEC ∆*gfcA* and EHEC wild-type strains were more susceptible to HD-5 than EPEC wild-type. Since the G4C is composed of the same sugar repeats as the lipopolysaccharide O-antigen, an -antigen ligase (*waaL*) deletion mutant was generated in EPEC to assess its role in HD-5 resistance. The ∆*waaL* EPEC strain was more susceptible to HD-5 than both the wild-type and ∆*gfcA* strains. The ∆*gfcA*∆*waaL* EPEC strain was not significantly more susceptible to HD-5 than the ∆*waaL* strain, suggesting that the absence of -antigen influences G4C formation. To determine whether the G4C and -antigen interact with HD-5, total polysaccharide was purified from wild-type EPEC and added to the ∆*gfcA*∆*waaL* strain in the presence of HD-5. The addition of exogenous polysaccharide protected the susceptible strain against HD-5 killing in a dose-dependent manner, suggesting that HD-5 binds to the polysaccharides present on the surface of EPEC. Altogether, these findings indicate that EPEC relies on both the G4C and the -antigen to resist the bactericidal activity of HD-5.

## Introduction

Enteropathogenic *Escherichia coli* (EPEC) is one of the leading causes of infant diarrheal morbidity and mortality in developing countries [1,2]. Enterohemorrhagic *Escherichia coli* (EHEC) is a genetically related pathogen that causes food-borne outbreaks of severe diarrhea in developed countries [3,4]. Both EPEC and EHEC cause histopathological lesions known as attaching and effacing (A/E) lesions, characterized by the localized effacement of microvilli, the intimate attachment of bacteria to the enterocyte plasma membrane and the formation of pedestal-like structures beneath sites of bacterial attachment [5,6]. A/E pathogens carry the pathogenicity island known as the locus of enterocyte effacement, which is required for A/E lesion-formation. Despite similarities in virulence factors, EPEC and EHEC have strict tissue tropism for the human small intestine and colon, respectively [3,7]. Intimate adherence to the intestinal mucosa exposes these pathogens to secreted antimicrobial peptides (AMPs).

AMPs are critical components of the innate immune system. These short (~20-50 amino acids) and cationic peptides are involved in host defense through both direct bactericidal and immunomodulatory properties [8,9]. Mammalian AMPs are grouped into two major families, the cathelicidins and the defensins. LL-37 is the sole human cathelicidin; it is expressed by a variety of cell types including neutrophils and epithelial cells. Defensins are further divided into α- and β-defensins, based on different disulfide bridge connectivity. Six α-defensins have been characterized in humans [10]. There are four neutrophil-derived peptides (HNP 1-4) and the enteric α-defensins (HD-5 and -6), the latter are mainly produced by Paneth cells in the small intestine. Although many genes encode human β-defensins, only four (hBD 1-4) have been well characterized; they are expressed in a constitutive or inducible manner by epithelial cells. Cationic AMPs interact with negatively charged bacterial membranes through electrostatic interactions. AMPs lyse bacterial cells by forming pores in the cytoplasmic membrane and/or by targeting key bacterial processes, such as cell wall synthesis [11].

Bacterial pathogens have evolved different mechanisms to survive the bactericidal activity of AMPs. For example, Gram-negative bacteria produce proteases that degrade AMPs, they down-regulate the expression of AMPs by host cells, they covalently modify their lipopolysaccharide (LPS) to prevent AMP-binding, or they produce surface structures such as capsule polysaccharides that shield the cell surface [12]. The contribution of each of these mechanisms for a given pathogen remains to be specified. Capsule polysaccharides and LPS O-antigens are recognized bacterial virulence factors that have been associated with AMP resistance [13,14]. For example, anionic capsules were shown to bind cationic AMPs to promote bacterial resistance [15]. In addition to capsules, some O-antigens have been shown to contribute to AMP resistance [16,17]. In *Klebsiella pneumoniae*, the capsule confers resistance against airway defensins, but the LPS O-antigen is dispensable for resistance [18]. Therefore, the contribution of structures that shield the bacterial surface appears to vary between species.

In *E. coli*, capsule polysaccharides are divided into 4 groups [14]. Extraintestinal *E. coli* isolates typically express group 2 or 3 capsules, whereas pathogenic intestinal *E. coli* isolates can express group 1 or 4 capsules. The capsule polysaccharide produced by both EPEC and EHEC belongs to the fourth group and is called the group 4 capsule (G4C). The G4C is composed of the same sugar repeats as the LPS O-antigen [14,19]. The machinery for the production of the sugars comprising the capsular polysaccharide and LPS O-antigen is shared. In the prototypical EPEC strain E2348/69 the G4C and O-antigen consist of repeats of a linear tetrasaccharide made of L-fucose, D-galactose and two *N*-acetyl-galactosamines [20,21]. In EHEC O157 strains, this linear tetrasaccharide is made of guanosine diphosphate-4-acetamido-D-rhamnose, L-fucose, D-glucose, and *N*-acetyl-D-galactosamine [22,23]. The export machinery for the G4C in EPEC and EHEC is encoded by the *gfc* operon [19]. All genes in the *gfc* operon have been identified as being essential for capsule secretion and the crystal structures of some proteins have been elucidated [19,24]. The role of the G4C during EPEC and EHEC colonization remains unclear. Previous studies showed temporal regulation of the G4C during infection; the G4C is produced during early contacts with eukaryotic cells and is downregulated at later time points to allow direct contact between the type III secretion system and epithelial cells [25]. To date, no study has addressed the role of G4Cs in AMP resistance.

During infection, EPEC predominantly colonizes the human proximal small intestine [3,7]. Paneth cells of the human small intestine secrete numerous antimicrobial components, including lysozyme, phospholipase A_2_, RegIIIα, and the enteric α-defensins HD-5 and HD-6, which are the most abundant antimicrobial compounds in Paneth cells [26,27]. HD-5 and HD-6 are constitutively expressed and stored as inactive precursors in Paneth cell secretory granules. After stimulation by bacteria, the contents of the granules are released into the intestinal crypts and the inactive pro-peptides are processed by host proteases into their active mature forms. Transgenic mice expressing either human HD-5 or HD-6 are resistant to *Salmonella* infection [28,29]. However, HD-5 and HD-6 appear to have different mechanisms of action. HD-5 has potent bactericidal activity [30], whereas HD-6 is mostly devoid of bactericidal activity but appears to form nets surrounding *Salmonella* cells to prevent invasion of host cells [29]. In addition, transgenic mice expressing HD-5 had altered microbiota composition, whereas those expressing HD-6 did not exhibit significant changes [29,31]. In contrast to HD-5 and HD-6, β-defensins and LL-37 are poorly expressed in the human small intestine [32,33].

Previously, we have shown that the *E. coli* outer-membrane protease OmpT cleaves and inactivates LL-37, although OmpT cannot cleave oxidized defensins [34,35]. Strikingly, OmpT was expressed at lower levels in EPEC compared to EHEC, suggesting that EPEC relies on other mechanisms to resist the AMPs present in the small intestine [34]. In this study, we assessed the contribution of EPEC surface structures in HD-5 resistance, the most abundant and bactericidal AMP in the small intestine. Our results show that both the EPEC G4C and LPS O-antigen play important roles in HD-5 resistance, most likely by interacting with HD-5.

## Results

### Surface Structures Expressed by EPEC

**Figure 1:**
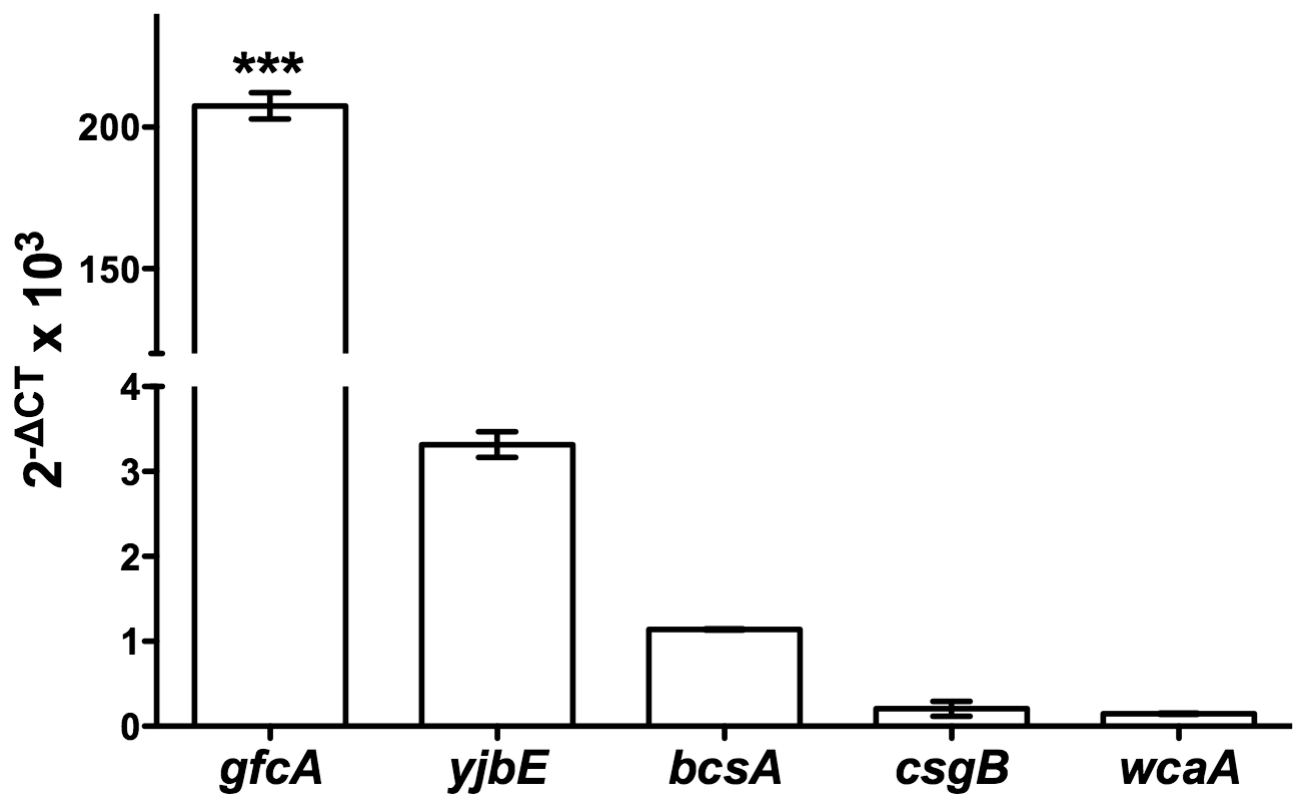
Expression of surface structures by EPEC. Transcription of the indicated genes was quantified by qPCR. Data shown (2^-ΔCT^ x 10^3^) are normalized against transcription of the 16S RNA gene. Results are expressed as means ± SEs of triplicate samples. Asterisks indicate statistical significance; ***, *P* <0.001 by one-way ANOVA and Bonferroni’s multiple comparison *post* *hoc* test.

Bacterial surface structures such as capsules, exopolysaccharides and curli have been implicated in AMP resistance [12]. To identify the surface structures expressed by EPEC cells grown in N-minimal medium, the transcript levels of the genes required for production of G4C (*gfcA*), exopolysaccharide (*yjbE*) [36], cellulose (*bcsA*), curli (*csgB*), and colanic acid (*wcaA*) were measured by qPCR. Transcript levels of *gfcA* were approximately 70-fold higher than those observed for any other gene assayed (Figure 1). This indicates that the G4C is likely produced by EPEC under these experimental conditions. 

### EPEC and EHEC Differentially Express the G4C

**Figure 2:**
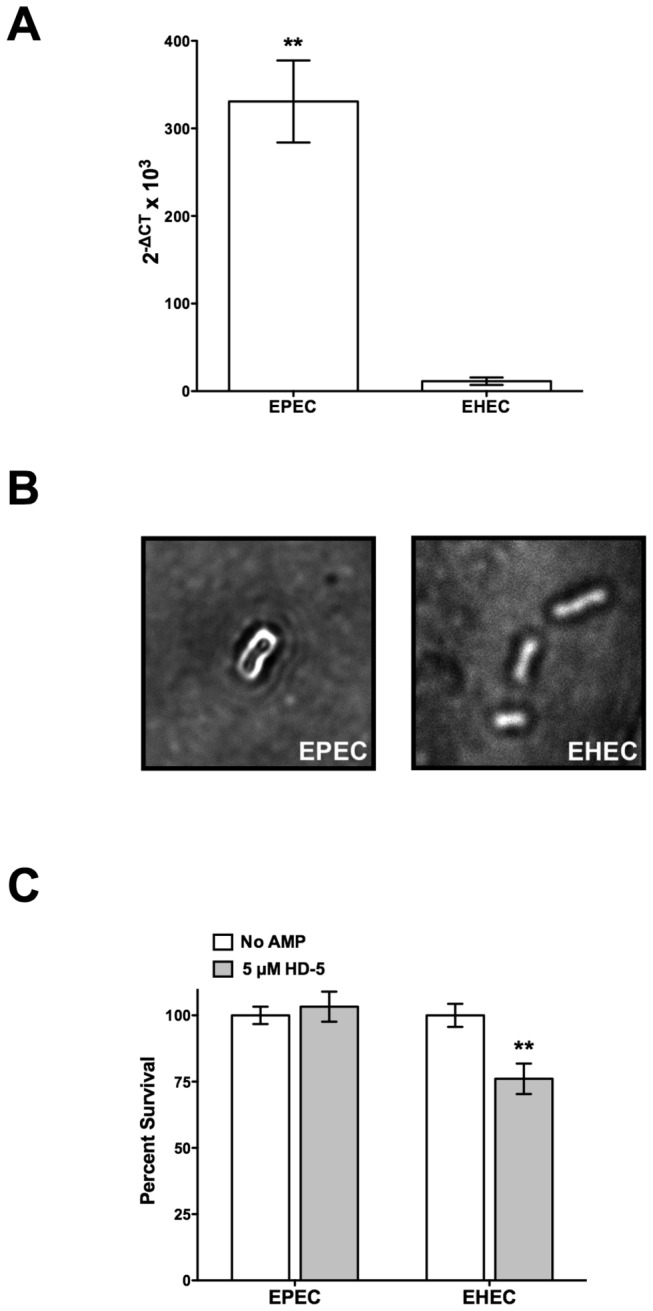
Capsule production and HD-5 resistance in EPEC and EHEC. (A) Transcription of *gfcA* in the EPEC and EHEC wild-type strains was quantified by qPCR. Data shown (2^-ΔCT^ x 10^3^) are representative of *gfcA* gene expression normalized against 16S RNA gene expression. Results are expressed as means ± SEs of triplicate samples. Asterisks indicate statistical significance; **, *P* <0.01 by paired *t* test. (B) Capsule staining of EPEC and EHEC wild-type strains, capsules are visualized by negative staining at a magnification of 100 X. Images shown are representative of at least ten fields of view from three independent experiments. (C) Survival of EPEC and EHEC wild-type cells in the presence of 5 µM HD-5. Results are expressed as means ± SEs of triplicate samples. Data shown are representative of at least three independent experiments. Asterisks indicate statistical significance; **, *P* <0.01 by two-way ANOVA and Bonferroni's multiple comparison *post* *hoc* test.

Both EPEC and EHEC have the genes encoding an active G4C export operon [19]. The expression of the first gene in the *gfc* operon, *gfcA* (also known as *ymcD* in EHEC), was measured by qPCR from EPEC and EHEC cells grown in N-minimal medium. The *gfcA* transcript levels in EHEC were approximately 30-fold lower than those found in EPEC (Figure 2A). To determine whether the differential *gfcA* expression correlates with capsule formation, capsule stains were performed. As shown in Figure 2B, EPEC produces a capsule as evidenced by the exclusion of staining around the bacterial cell. In contrast, no stain exclusion was observed around EHEC cells, suggesting that EHEC does not produce a capsule under these experimental conditions. To determine whether the presence of the capsule influences HD-5 resistance, the susceptibility of EPEC and EHEC to HD-5 was assessed. In the presence of HD-5, no change in survival was observed for EPEC, whereas EHEC cells showed a 25% decrease in survival (Figure 2C). These data indicate that high transcript levels of *gfcA* correlate with the presence of a capsule in EPEC. Although the capsular polysaccharide composition is different in EPEC and EHEC, the fact that EPEC is unaffected by HD-5 may suggest a role for the capsule in HD-5 resistance.

### Characterization of the *gfcA* Deletion Mutant in EPEC

To analyze the contribution of the G4C formed by EPEC in AMP resistance, an isogenic *gfcA* deletion mutant was generated in EPEC E2348/69. The ∆*gfcA* strain was complemented with the pACYC184-derived p*gfcA* plasmid [∆*gfcA*(p*gfcA*)]. These strains were assessed for the presence of the capsule by performing buoyancy assays and capsule staining. Differences in buoyancy on Percoll-step gradients are used to detect non-mucoid bacterial capsules, such as the G4C produced by EPEC [19,37]. Bacterial buoyancy is assessed by the formation of a band at the Percoll interface after low-speed centrifugation for encapsulated strains and formation of a pellet for unencapsulated strains. As expected, the ∆*gfcA* strain formed a pellet, indicating lower buoyancy and the absence of capsule. The wild-type and ∆*gfcA*(p*gfcA*) strains were found at the Percoll interface, indicating higher buoyancy and the presence of a capsule. Capsule staining confirmed that the ∆*gfcA* strain did not form a capsule and that plasmid complementation of the ∆*gfcA* strain restored capsule formation (data not shown). These data identify the capsule produced by wild-type EPEC in N-minimal medium as the G4C.

### The G4C Promotes Resistance to HD-5

**Figure 3:**
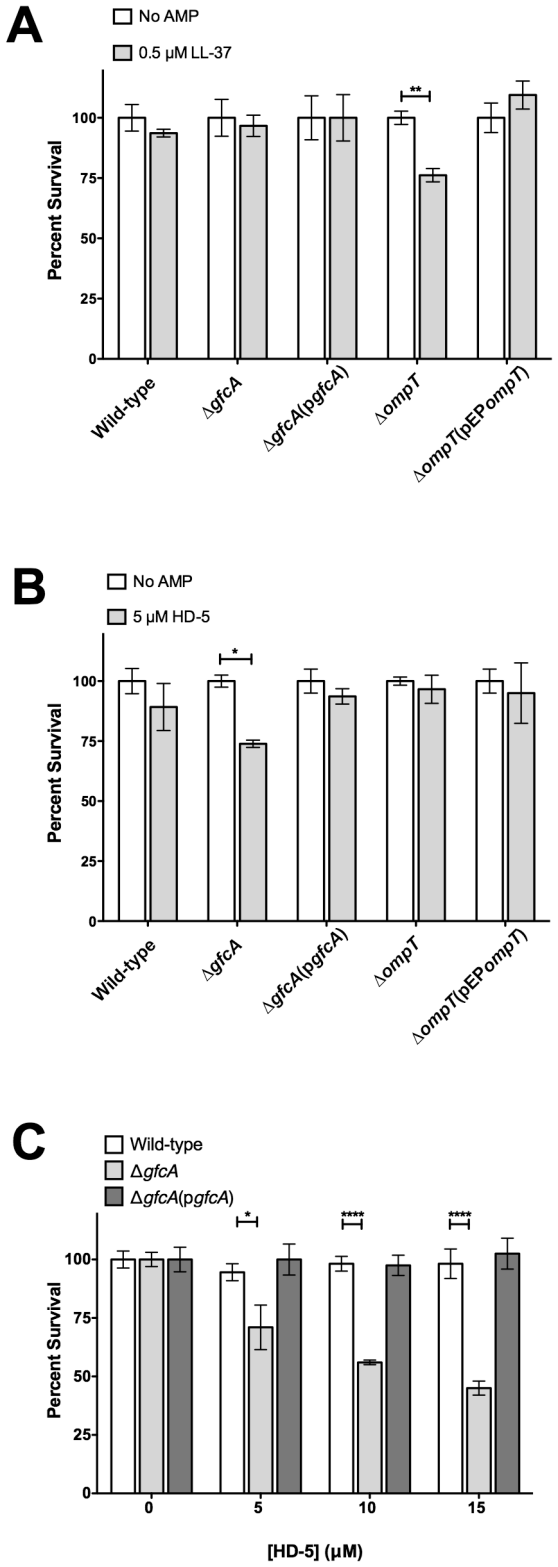
The G4C promotes resistance to HD-5. Survival of the indicated EPEC strains in the presence of LL-37 (A) or in the presence of HD-5 (B and C) at the indicated concentrations. Results are expressed as means ± SEs of triplicate samples. Data shown are representative of at least three independent experiments. Asterisks indicate statistical significance; *, *P*<0.05; **, *P*<0.01; ****, *P*<0.0001 by two-way ANOVA and Bonferroni’s multiple comparison *post* *hoc* test.

To assess the contribution of the EPEC G4C to AMP resistance, the susceptibility of the wild-type, ∆*gfcA* and ∆*gfcA*(p*gfcA*) strains to LL-37 and HD-5 was determined. Due to the low abundance of LL-37 in the small intestine, we used a concentration of 0.5 µM in our assays. The wild-type, ∆*gfcA* and ∆*gfcA*(p*gfcA*) strains were not susceptible to LL-37 (Figure 3A). The ∆*ompT* strain that was previously reported to be unable to inactivate LL-37 was used as a control [34]. This strain showed a ~25% decrease in survival when compared to wild-type (Figure 3A), confirming that this concentration of LL-37 is sufficient to kill susceptible strains. As expected, complementation of ∆*ompT* with pEP*ompT* restored survival to wild-type levels (Figure 3A). These data indicate that the G4C is dispensable for LL-37 resistance. 

During bacterial infection, HD-5 is estimated to be present in the human small intestine at concentrations of 1-5 µM [38]. We tested the susceptibility of the ∆*gfcA* strain to physiological concentrations of HD-5 (1-5 µM). Concentrations above 2 µM resulted in some level of killing; however, the killing was only statistically significant at a concentration of 5 µM (data not shown). Incubation with 5 µM HD-5 resulted in a 25% decrease in survival for the ∆*gfcA* strain (Figure 3B). In contrast, no significant changes were observed for the wild-type, ∆*ompT* and complemented strains in the presence of 5 µM HD-5 (Figure 3B). Interestingly, similar susceptibility to HD-5 was observed for the EPEC ∆*gfcA* (Figure 3B) and EHEC wild-type strains (Figure 2C). To determine if higher concentrations of HD-5 would increase the killing of these EPEC strains, survival assays were performed with 5 to 15 µM HD-5. The wild-type and ∆*gfcA*(p*gfcA*) strains were unaffected by HD-5 even at a concentration of 15 µM (Figure 3C). In contrast, the ∆*gfcA* strain showed a significant dose-dependent decrease in survival. These data indicate that the G4C contributes to HD-5 resistance at the physiological concentration of 5 µM. 

### The O-antigen Also Contributes to HD-5 Resistance

**Figure 4:**
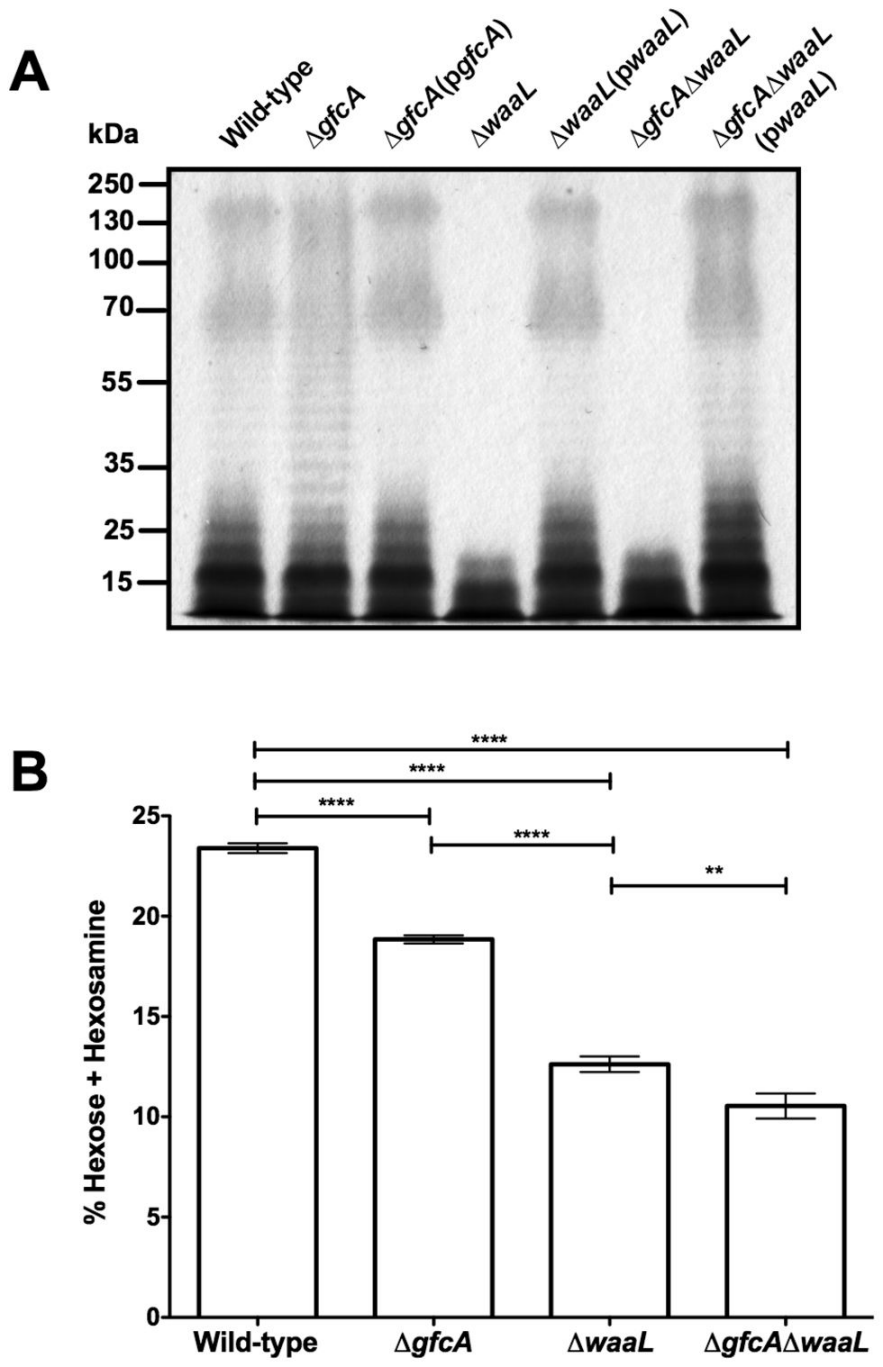
Interplay between G4C and O-antigen. (A) Silver staining of proteinase K-treated LPS of the indicated EPEC strains. All samples were normalized (by OD_600_) to ensure that the same number of cells was used. Data shown are representative of three independent experiments. (B) Percent of total combined hexose and hexosamine in each purified polysaccharide preparation ([mg hexose +hexosamine]/ mg purified polysaccharide) from the indicated strain. Results are expressed as mean ± SDs of samples. Asterisks indicate statistical significance; **, *P*<0.01; ****, *P*<0.0001 by one-way ANOVA and Bonferroni’s *post* *hoc* comparison test.

Because the G4C is known to have the same composition as the LPS O-antigen, we hypothesized that the O-antigen may also contribute to HD-5 resistance. To test this possibility, an O-antigen ligase (*waaL*) deletion mutant was generated in EPEC E2348/69 and the ∆*waaL* strain was complemented with the pACYC184-derived p*waaL* plasmid. The LPS profiles of the EPEC strains were compared after SDS-PAGE separation and LPS-specific silver staining. The LPS profile of wild-type EPEC showed a characteristic pattern of O-antigen banding (Figure 4A). The ∆*gfcA* strain showed a slightly different pattern with an increase in O-antigen laddering, indicating that the absence of capsule influences O-antigen length distribution. The wild-type LPS pattern was restored for the ∆*gfcA*(p*gfcA*) strain (Figure 4A). As expected, the ∆*waaL* and ∆*gfcA*∆*waaL* strains were devoid of O-antigen. Complementation of these strains with p*waaL* restored wild-type and ∆*gfcA* O-antigen laddering patterns, respectively (Figure 4A). Because the EPEC G4C does not migrate on an SDS-PAGE gel [19], capsule staining was performed on the ∆*waaL* strains to determine whether the absence of O-antigen affects capsule formation. The ∆*waaL* strain was found to contain a heterogeneous mixture of bacteria with and without G4C (data not shown). Complementation of ∆*waaL* with p*waaL* restored wild-type levels of encapsulation. To address the possibility that the ∆*waaL* strain is unencapsulated, we quantified the amount of C6 sugar [hexose (fucose and galactose) and hexosamine (*N*-acetyl-galactosamine)] from purified total polysaccharide. As expected, the ∆*gfcA* strain had less total C6 sugar than wild-type (Figure 4B). The ∆*waaL* strain had less total C6 sugar than the ∆*gfcA* strain, but more than the ∆*gfcA*∆*waaL* strain (Figure 4B). These findings indicate that the ∆*waaL* strain has some level of encapsulation. Altogether, these data are in agreement with previous studies that reported interplay between the G4C and the LPS O-antigen [14,19].

**Figure 5:**
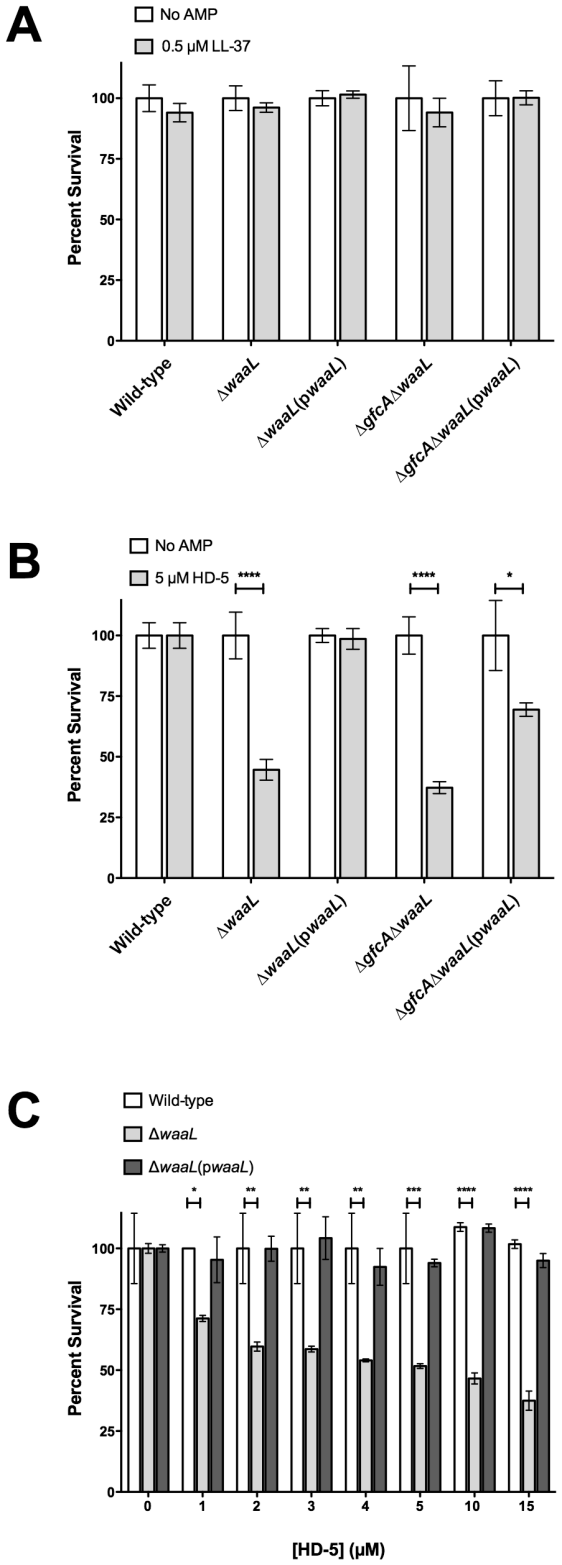
The O-antigen promotes resistance to HD-5. Survival of the indicated EPEC strains in the presence of LL-37 (A) and HD-5 (B and C) at the indicated concentrations. Results are expressed as means ± SEs of triplicate samples. Data shown are representative of at least three independent experiments. Asterisks indicate statistical significance; *, *P*<0.05; **, *P*<0.01; ***, *P*<0.001; ****, *P* <0.0001 by two-way ANOVA and Bonferroni’s multiple comparison *post* *hoc* test.

To determine the contribution of the EPEC O-antigen to AMP resistance, the susceptibility of the ∆*waaL* and ∆*gfcA*∆*waaL* strains to LL-37 and HD-5 was tested. When incubated with 0.5 µM LL-37, the ∆*waaL*, ∆*waaL*(p*waaL*), ∆*gfcA*∆*waaL*, and ∆*gfcA*∆*waaL*(p*waaL*) strains survived similarly to wild-type, indicating that the O-antigen does not contribute to LL-37 resistance (Figure 5A). When incubated with 5 µM HD-5, both the ∆*waaL* and ∆*gfcA*∆*waaL* strains showed at least a 50% decrease in survival (Figure 5B). Complementation of these strains with p*waaL* restored survival to wild-type and ∆*gfcA* levels, respectively (Figure 5B). As shown in Figure 5C, the ∆*waaL* strain showed a 29% decrease in survival when incubated with 1 µM of HD-5, indicating that the ∆*waaL* strain is more susceptible to HD-5 than the ∆*gfcA* strain. Further increasing the concentration of HD-5 (3 to 15 µM) gradually reduced survival of the ∆*waaL* strain between 30-60% (Figure 5C). The wild-type and ∆*waaL*(p*waaL*) strains consistently survived at all concentrations of HD-5 assayed (Figure 5C). Taken together, these data indicate that both the O-antigen and the G4C contribute to HD-5 resistance.

### HD-6 does Not Enhance the Bactericidal Activity of HD-5

**Figure 6:**
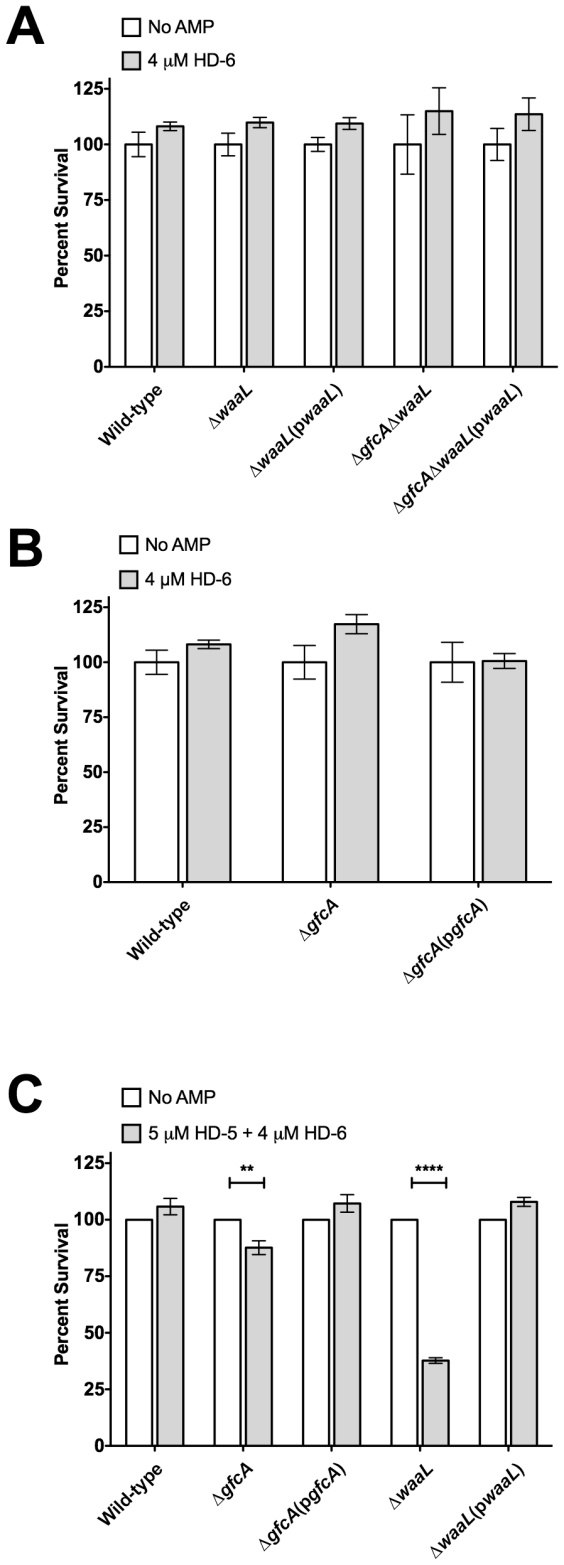
HD-5 and HD-6 do not synergize. Survival of EPEC wild-type, ∆*gfcA*, ∆*gfcA*(p*gfcA*) strains (A) and EPEC wild-type, ∆*waaL*, ∆*waaL*(p*waaL*), ∆*gfcA*∆*waaL* and ∆*gfcA*∆*waaL*(p*waaL*) strains (B) in the presence of 4 µM HD-6. (C) Survival of EPEC wild-type, ∆*gfcA*, ∆*gfcA*(p*gfcA*), ∆*waaL* and ∆*waaL*(p*waaL*) in the presence of 5 µM HD-5 and 4 µM HD-6. Results are expressed as means ± SEs of triplicate samples. Data shown are representative of at least three independent experiments. Asterisks indicate statistical significance; **, *P*<0.01; ****, *P* <0.0001 by one-way ANOVA and Bonferroni’s multiple comparison *post* *hoc* test.

HD-6 is the second most abundant AMP in the small intestine [39]. We tested the antimicrobial activity of HD-6 against the EPEC ∆*gfcA*, ∆*waaL*, and ∆*gfcA*∆*waaL* strains. None of the strains were susceptible to 4 µM HD-6 (Figure 6A and B). These results are consistent with previous studies that have shown that HD-6 is not bactericidal against *E. coli* [30]. Because HD-6 has been shown to bind bacterial surfaces and form fibrils that entangle bacteria [29], we tested whether the presence of HD-6 enhances the bactericidal activity of HD-5. As shown in Figure 6C, the ∆*gfcA* and ∆*waaL* strains showed ~30% and ~50% decrease in survival, respectively, and the wild-type and complemented strains were unaffected. These data are similar to what was observed when the bacteria were incubated with HD-5 alone, indicating the absence of synergy between HD-5 and HD-6.

### The Addition of Exogenous EPEC Polysaccharide Protects Against HD-5 Killing

**Figure 7:**
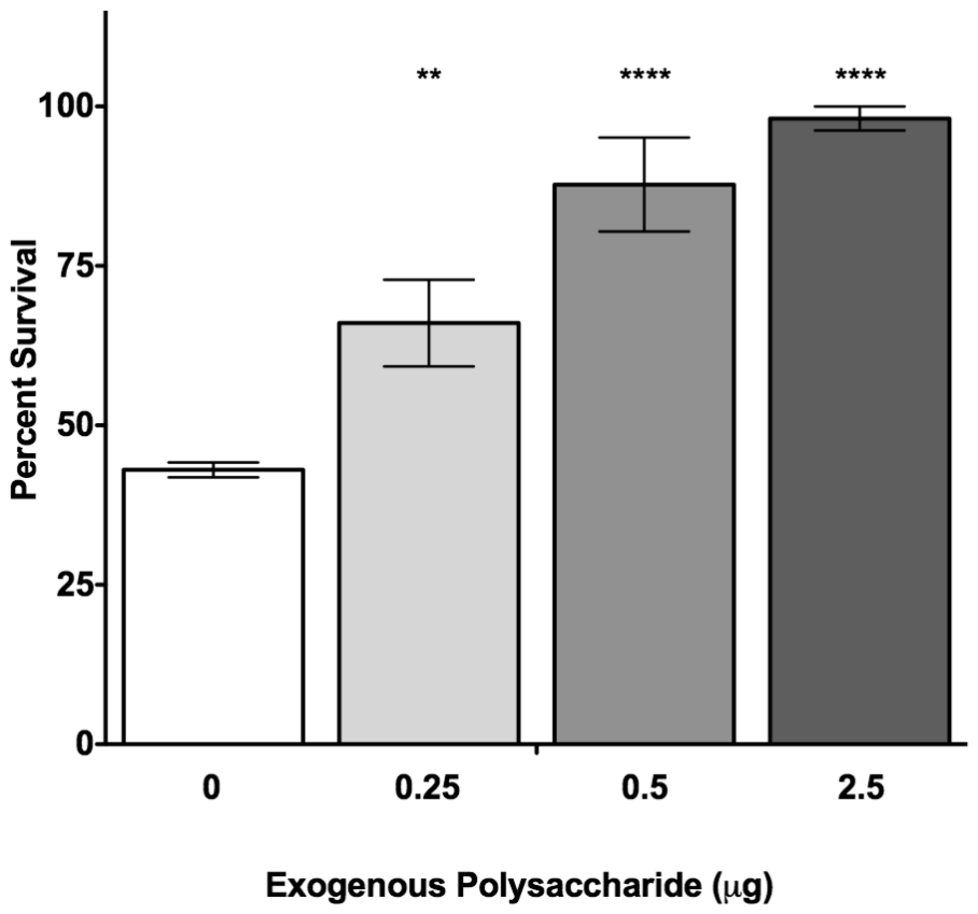
Exogenous EPEC polysaccharide protects against HD-5 killing. Survival of EPEC ∆*gfcA*∆*waaL* cells in the presence of 5 mM HD-5 supplemented with the indicated amount of EPEC polysaccharide purified from wild-type cells. The control does not have exogenous polysaccharide added to the sample. Results are expressed as means ± SEs of triplicate samples. Data shown are representative of at least three independent experiments. Asterisks indicate statistical significance compared to the control group; **, *P* <0.01; ****, *P* <0.0001 by two-way ANOVA and Bonferroni’s multiple comparison *post* *hoc* test.

To investigate whether the G4C and O-antigen interact with HD-5 and prevent HD-5 from accessing the bacterial membrane, we measured survival of the ∆*gfcA*∆*waaL* strain in the presence of increasing amounts of total polysaccharide, containing both G4C and LPS O-antigen, purified from EPEC wild-type. As little as 0.25 µg of polysaccharide was able to reduce HD-5-mediated killing of the ∆*gfcA*∆*waaL* strain by ~20%, killing was further decreased by up to ~60% by the addition of increased amounts of polysaccharide (Figure 7). Therefore, the addition of exogenous polysaccharide protects against HD-5 killing in a dose-dependent manner. These data indicate that the purified polysaccharide interacts with HD-5, suggesting that the EPEC G4C and O-antigen trap HD-5 before it reaches the bacterial membrane.

## Discussion

Human α-defensins play a crucial role in protecting the small intestine mucosa from bacterial pathogens. HD-5 is the most abundant AMP released in the small intestine by Paneth cells [26,27]. EPEC, which has strict tissue tropism for the small intestine [3,7], likely encounters this AMP during colonization. The aim of this study was to identify the surface structures used by EPEC to resist HD-5 killing. Of the surface structure genes analyzed, *gfcA* was by far the most expressed (Figure 1). Therefore, we investigated its role in HD-5 resistance. The unencapsulated EPEC *gfcA* mutant was more susceptible to HD-5 than wild-type (Figure 3). Because the composition of the G4C is similar to that of the -antigen, we also investigated the role of the -antigen in HD-5 resistance. The EPEC *waaL* mutant was more susceptible to HD-5 than wild-type and the *gfcA* mutant (Figure 5). This study shows that both the EPEC G4C and -antigen are involved in HD-5 resistance.

HD-5 is constitutively expressed in the human small intestine. Mature HD-5 released in the small intestine is estimated to reach concentrations in the range of 1-5 µM [38]. Therefore, the HD-5 concentrations used in this study are consistent with those found in the small intestine. Both the EPEC *gfcA* and *waaL* mutants were susceptible to 5 µM HD-5, in contrast to wild-type that was unaffected. These *in vitro* data may suggest that both the G4C and -antigen play roles in HD-5 resistance *in vivo*. The second most abundant AMP in the small intestine, HD-6, is devoid of bactericidal activity [32,40]. HD-6 was added to HD-5 to assess if it could enhance HD-5-mediated killing of EPEC. No synergistic effects were observed when HD-5 and HD-6 were combined (Figure 6). However, it remains possible that other antimicrobial components present in the small intestinal lumen, such as lysozyme or lactoferrin, act in combination with HD-5 and/or HD-6 to influence EPEC colonization. LL-37 is poorly expressed in the human small intestine [33]. At a concentration of 0.5 µM, the *gfcA* and *waaL* mutants were unaffected by LL-37 (Figures 3 and 5), suggesting they do not play a major role in LL-37 resistance. As expected, OmpT did not contribute to HD-5 resistance (Figure 3), likely due to its disulfide bridges that render it resistant to protease cleavage [35,41]. Together, our data suggest that EPEC relies mainly on surface structures to resist the bactericidal activity of enteric α-defensins.

EPEC expresses a G4C, which is far less studied than the other groups of capsule. To our knowledge, this is the first study showing the involvement of a G4C in AMP resistance. It was previously proposed that anionic capsules better protect bacteria from cationic AMPs [15]. The EPEC O127 G4C is made of repeats of a neutral tetrasaccharide [20,21]. Therefore, our results suggest that the presence of anionic charges is dispensable to the protective action of the G4C against HD-5. This conclusion is in good agreement with the fact that HD-5 is known to have lectin-like properties and bind glycoproteins of viral envelopes [42]. In support, the EPEC G4C is also protective against another α-defensin with known lectin properties, Human Neutrophil Peptide-1 (HNP-1) (Thomassin et al., unpublished data). 

This study shows that the *waaL* mutant is more susceptible to HD-5 than the *gfcA* mutant (Figures 3 and 5). One would be tempted to conclude that the O-antigen plays a more important role than the G4C in HD-5 resistance. However, LPS analysis of the *gfcA* mutant revealed that the absence of the G4C affects the length distribution of the O-antigen (Figure 4A). These changes in LPS have been related to serum resistance [19,43]. However, it remains unclear whether the altered O-antigen length distribution affects HD-5 resistance. Conversely, the absence of O-antigen appeared to affect G4C formation. The heterogeneity in G4C formation of the ∆*waaL* strain and the small (2%) difference in total C6 sugar content between the ∆*waaL* and ∆*gfcA*∆*waaL* strains may be caused by the absence of K_LPS_, the portion of the capsular polysaccharide that is attached to the lipid A-core, in the ∆*waaL* strain [44]. These data are in good agreement with previous reports that suggest interplay between the G4C and the O-antigen [19,43]. Consequently, we conclude that it is the total amount of polysaccharide, containing both G4C and LPS O-antigen, present on the surface of EPEC that is important for HD-5 resistance. The correlation between the survival of the ∆*gfcA*∆*waaL* strain and the amount of exogenous polysaccharide added to the assay strongly supports this conclusion (Figure 7).

This study shows that greater G4C production by EPEC than EHEC correlates with increased HD-5 resistance in EPEC. Previously, we reported that higher OmpT levels in EHEC than EPEC were related to increased LL-37 resistance in EHEC [34,35]. Altogether, these studies suggest that EPEC and EHEC have evolved to differentially express the genes responsible for the resistance mechanisms that protect them from the AMPs present in their respective niches.

## Materials and Methods

### Media and Reagents

Bacteria were grown at 37C with aeration (200 rpm) in Luria-Bertani (LB) broth or N-minimal medium adjusted to pH 7.5 and supplemented with 0.2% glucose and 1 mM MgCl_2_. When appropriate, media were supplemented with ampicillin (Amp; 100 μg/ml), streptomycin (Strep; 50 μg/ml), or chloramphenicol (Cm; 30 μg/ml). LL-37 was synthesized with a purity of > 85% (BioChemia). HD-5 and HD-6 were purchased from Peptides International Inc. AMPs were reconstituted in sterile dH_2_O. Restriction enzymes were from New England Biolabs and iProof DNA Polymerase was from Bio-Rad.

### Construction of Deletion Mutants

The bacterial strains and plasmids used in this study are listed in Table 1. DNA purification, cloning, and transformation were performed according to standard procedures [45]. The EPEC ∆*gfcA* and ∆*waaL* strains were generated by *sacB* gene-based allelic exchange [46]. Genomic DNA from EPEC was used as a template to PCR-amplify the upstream sequences (primer pairs gfcA1gfcA2 or waaL1waaL2) (Table 2) and downstream sequences (primer pairs gfcA3gfcA4 or waaL3waaL4) of the *gfcA* or *waaL* genes. The resultant PCR products were treated with the appropriate restriction enzyme (Table 2) and ligated together. The ligation products were then used as the DNA templates in PCR reactions with the primers gfcA1gfcA4 or waaL1waaL4. PCR products were gel-purified, digested with the appropriate restriction enzymes and ligated into pRE112 cleaved with either XbaISacI or XbaIKpnI. Resultant plasmids p∆*gfcA* and p∆*waaL* were verified by sequencing at the McGill University and Genome Québec Innovation Centre. The p∆*gfcA* and p∆*waaL* constructs were introduced into wild-type EPEC by conjugation using *E. coli* Sm10 (λ Pir) as the donor strain; integration of the plasmid into the chromosome was selected for by plating bacteria on LB agar supplemented with Cm and Strep. Cm-resistant transformants of EPEC were then cultured on peptone agar containing 5% sucrose to isolate sucrose-resistant colonies. To confirm excision of the suicide vector, sucrose-resistant colonies were tested for Cm sensitivity. Gene deletions were verified by PCR. The ∆*gfcA*∆*waaL* strain was generated by transforming p∆*waaL* into the ∆*gfcA* strain and performing *sacB*−gene based allelic exchange, as described above.

**Table 1:** Bacterial strains and plasmids used in this study.

Strain or plasmid	Description	Reference/source
Strains		
EHEC EDL933	Wild-type EHEC O157:H7	[53]
EPEC E2348/69	Wild-type EPEC O127:H6, Str**^*r*^**	[54]
EPEC ∆*ompT*	E2348/69 ∆*ompT*	[34]
EPEC ∆*ompT*(p*ompT*)	E2348/69 ∆*ompT* expressing *ompT* from p*ompT*	[34]
EPEC ∆*gfcA*	E2348/69 ∆*gfcA*	This study
EPEC ∆*gfcA*(p*gfcA*)	E2348/69 ∆*gfcA* expressing *gfcA* from p*gfcA*	This study
EPEC ∆*waaL*	E2348/69 ∆*waaL*	This study
EPEC ∆*waaL*(p*waaL*)	E2348/69 ∆*waaL* expressing *waaL* from p*waaL*	This study
EPEC ∆*gfcA*Δ*waaL*	E2348/69 ∆*gfcA*∆*waaL*	This study
EPEC ∆*gfcA*∆*waaL*(p*waaL*)	E234869 ∆*gfcA*Δ*waaL* expressing *waaL* from p*waaL*	This study
Sm10 (λPir)	*thi thr leuB tonA lacY supE recA*::RP4-2-Tc::Mu-Kan Kan**^*r*^**	
Sm10 (λPir)(p∆*gfcA*)	Sm10 (λPir) containing p∆*gfcA*	This study
Sm10 (λPir)(p∆*waaL*)	Sm10 (λPir) containing p∆*waaL*	This study
Plasmids		
pRE112	Sucrose sensitive (*sacB*1) suicide vector, Cm^r^	[55]
p∆*gfcA*	EPEC ∆*gfcA* deletion construct in pRE112	This study
p∆*waaL*	EPEC ∆*waaL* deletion construct in pRE112	This study
pACYC184	Cloning vector, Tetracycline**^*r*^** Cm^r^	NEB
pEP*ompT*	EPEC *ompT* cloned into pACYC184	[34]
p*gfcA*	EPEC *gfcA* cloned into pACYC184	This study
p*waaL*	EPEC *waaL* cloned into pACYC184	This study

**Table 2:** Primers used in this study.

Primer	Sequence^*a*^	Usage
gfcA1	CTAGTCTAGAGTATGCTGTCTGTCTTTCAAACCGAC	∆*gfcA* 5’F XbaI
gfcA2	GCCGGTACCCATAACTTTTCCTTTATTCATC	∆*gfcA* 5′R KpnI
gfcA3	GCCGGTACCTAACTACGCGCTAATACCACTTTAACG	∆*gfcA* 3’F KpnI
gfcA4	CTAGGAGCTCCACCATAACGTTATTTTTGTCC	∆*gfcA* 3’R SacI
gfcA5	GGGCTCTAGATATGACGCTGCTTGTTTAAAACTG	p*gfcA* F XbaI
gfcA6	CTTATGAGCTCTTAGCGCGTAGTGGATGTGGTG	p*gfcA* R SacI
waaL1	TGCTCTAGACGATGTTCTCCATACGTTGA	∆*waaL* 5’F XbaI
waaL2	CCGCTCGAGCATTGCTTCTCCACCATCTT	∆*waaL* 5’R XhoI
waaL3	CCGCTCGAGGGTTTGCTGTTAGCTATGAG	∆*waaL* 3’F XhoI
waaL4	GCAGGTACCCGCATGTATTCGAAACGAGG	∆*waaL* 3’R KpnI
waaL5	GCACCGATATCCACGTTCTATATTATTAAGATGG	p*waaL* F EcoRV
waaL6	CCCGGATCCTGCCTGAACTCAATGTCAG	p*waaL* R BamHI
qEH16SF	GTGCTGCATGGCTGTCGTCA	qPCR EHEC 16S F
qEH16SR	AGCACGTGTGTAGCCCTGGT	qPCR EHEC 16S R
qEHgfcAF	GAGAACCGCCATCGCCACTG	qPCR EHEC *gfcA* F
qEHgfcAR	CAACCACGACGGCTGCTACC	qPCR EHEC *gfcA* R
qEP16SF	AACGCGTTAAGTCGACCGCC	qPCR EPEC 16S F
qEP16SR	CGGCTCCCGAAGGCACATTC	qPCR EPEC 16S R
qEPgfcAF	GCAACAACTGCCAACGTCGC	qPCR EPEC *gfcA* F
qEPgfcAR	CCACTACCGGTGCAACCACG	qPCR EPEC *gfcA* R
qEPyjbEF	GTCAGCACCGTAAGCTCGGC	qPCR EPEC *yjbE* F
qEPyjbER	CTGGGTACTGGTGGTCGTGGT	qPCR EPEC *yjbE* R
qEPbcsAF	TGTATCCGCACGGGCAAACG	qPCR EPEC *bcsA* F
qEPbcsAR	GGCCCGCGTGATGGGTAATG	qPCR EPEC *bcsA* R
qEPcsgBF	TGCAGCCGCAGCAGGTTATG	qPCR EPEC *csgB* F
qEPcsgBR	TCTTGCGCAACAACCGCCAA	qPCR EPEC *csgB* R
qEPwcaAF	ATGGGCGAGGAAGACGCTCA	qPCR EPEC *wcaA* F
qEPwcaAR	ACATTAACAGCGGGGCGTGC	qPCR EPEC *wcaA* R

^*a*^ Restriction sites are underlined; F indicates forward and R indicates reverse

The p*gfcA* complementation plasmid was constructed by PCR-amplifying the *gfcA* gene and its promoter from EPEC genomic DNA using primers gfcA5 and gfcA6 (Table 2). The resultant PCR product was cloned into the XbaI and EcoRV restriction sites of plasmid pACYC184, generating plasmid p*gfcA*. Similarly, the p*waaL* plasmid was generated by PCR-amplifying the *waaL* gene and a few base pairs upstream of the ATG using primers waaL5 and waaL6. The resultant DNA fragment was treated with EcoRV and BamHI and ligated downstream of the tetracycline promoter of pACYC184.

### Quantitative PCR

Quantitative PCR (qPCR) was performed as previously described [34]. Briefly, EPEC and EHEC strains were grown to an OD_600_ of 0.5 in N-minimal medium. Total RNA was isolated using TRIzol reagents (Invitrogen) and treated with the DNA-free kit (Ambion) to remove any remaining DNA. The absence of contaminating DNA was confirmed by qPCR using primers qEP16SF/qEP16SR or qEH16SF/qEH16SR (Table 2). RNA (1 μg) was reverse-transcribed using Superscript II (Invitrogen) with 0.5 μg of random hexamer primers (Sigma). As a negative control, a reaction without Superscript II was also included. qPCR reactions were performed in a Rotor-Gene 3000 thermal cycler (Corbett Research) by using the QuantiTect SYBR Green PCR kit (Qiagen), according to manufacturer's instructions. Primers used are listed in Table 2. The level of mRNA gene transcript was normalized to 16S RNA and analyzed using the 2^-∆CT^ method [47]. qPCR reactions were performed from three independent reverse transcription reactions.

### Capsule Stain

Bacterial cells were grown to an OD_600_ of 0.5 in N-minimal medium. Bacterial cultures (20 μl) were mixed with a drop of nigrosin (10% [w/v] nigrosin, Sigma), spread on a glass slide and allowed to air dry. Smears were fixed with ethanol for 2 min. Cells were then stained with crystal violet (2% [w/v] crystal violet, 20% [v/v] ethanol, 0.2% ammonium oxalate) for 2 min. Slides were washed, air dried and preserved with Cytoseal (Thermo Scientific) and glass coverslips. Slides were visualized under an oil immersion 100 x objective using a Nikon Eclipse Ti inverted microscope at the Centre for Microscopy at Concordia (CMAC, Concordia University). Images were captured with an Andor Neo sCMOS camera. The presence of a capsule was indicated by the exclusion of nigrosin and crystal violet [48]. 

### Survival Assays

Bacterial survival assays were performed as previously described with modifications [49]. Bacterial cells were grown to an OD_600_ of 0.5 in N-minimal medium, diluted to 6 x 10^4^ colony-forming units (CFU)/ml and aliquoted. The concentration of bacteria at the beginning of the experiment was verified by serial dilution and CFU enumeration the next day. An equal volume of AMP or N-minimal medium was added to the bacterial aliquots and tubes were incubated for 1 h at 37°C. After incubation, samples were serially diluted in N-minimal medium, plated onto LB-agar and incubated overnight at 37°C. Colonies were enumerated the next day. Percent survival is shown as 100x[(CFU/ml treated)/(CFU/ml untreated)]. 

### Buoyancy Assays

Buoyancy assays were performed as previously described with minor modifications [19]. Bacteria were grown in conical tubes in 5 ml N-minimal medium without shaking at 37°C until an OD_600_ of 0.5 was reached. Cultures were underlayed with 2 ml of Percoll [55% Percoll (v/v) in 25 mM phosphate buffer, pH 6.5] and centrifuged at 1,000 X *g* for 20 min. Encapsulated EPEC strains formed a band at the Percoll-medium interface, whereas strains without capsule formed pellets.

### Analysis of LPS

Bacterial cells were grown to an OD_600_ of 0.5 in N-minimal medium. Cells were concentrated 50 fold by centrifugation and resuspended in 2 x electrophoresis-sample buffer (0.0625 M Tris-HCl [pH 6.8], 1% [w/v] SDS, 10% glycerol, 2% [v/v] 2-mercaptoethanol, 0.001% [w/v] bromophenol blue). Samples were boiled for 10 min. Proteinase K (0.6 µg) was added and samples were incubated for 1 h at 60°C. Samples were frozen at -20°C until used. LPS species were separated by SDS-PAGE (10% acrylamide) and visualized by silver staining [50]. 

### Purification of Total Polysaccharide

Total polysaccharide was isolated from EPEC strains essentially as previously described [19]. Bacteria were grown in 100 mL of N-minimal medium with shaking at 37°C until an OD_600_ of 0.5 was reached. Cells were centrifuged at 4, 500 X *g* for 15 min, washed twice in phosphate-buffered saline (PBS, pH 7.4) and resuspended in a final volume of 0.5 ml PBS. An equal volume of saturated phenol (pH 8) was added, mixed and incubated at 70°C for 1 h with occasional mixing. The mixture was centrifuged for 1 h at 10, 000 X *g* and the top aqueous phase was collected. To remove contaminating protein, proteinase K (0.6 µg) was added and samples were incubated for 1 h at 60°C. Two volumes of 100% ethanol were added and samples were incubated at -70°C for 1 h. Samples were centrifuged for 30 min at 12, 000 X *g*, washed with 70% ethanol and air dried. Pellets were resuspended in dH_2_0 and aliquots were diluted to give a concentration approximately equivalent to 1 ml of cells at OD_600_ of 0.5 (5 µg/ml) prior to serial dilution for use in survival assays. 

### Quantitation of Sugar Content of Total Polysaccharide

Total hexose of capsule and LPS extracts were quantified by phenol-sulfuric acid method using a 1:1 galactose to fucose mixture as standard [51].  Total hexosamine was determined with p-(dimethylamino)-bezaldehyde reagent using galactosamine as standard after 4 h hydrolysis in 3 M trifluoroacetic acid at 100°C [52]. 
